# The spectrum-efficacy correlation of Kai-Xin-San for cognition of Aβ_42_ transgenic *Drosophila* and verification of its active ingredients

**DOI:** 10.3389/fphar.2025.1538837

**Published:** 2025-01-28

**Authors:** Jinfu Wu, Hang Sun, Yiyang Zhao, Lian Lian, Hongsheng Bian, Yong Guo, Dan Li, Lili Huang

**Affiliations:** ^1^ College of Pharmacy, Heilongjiang University of Chinese Medicine, Harbin, China; ^2^ Shineway Pharmaceutical Group Co., Ltd., Shijiazhuang, China

**Keywords:** Kai-Xin-San, spectrum-efficacy relationship, Alzheimer’s disease, pharmacodynamic material basis, HPLC, Aβ42 transgenic *Drosophila*

## Abstract

**Introduction:**

This study aims to establish the fingerprint spectra of Kai-Xin-San (KXS) and investigate its spectrum-effect relationship in treating Alzheimer’s disease (AD).

**Methods:**

Initially, the fingerprints of 15 batches of KXS were established and analyzed using HPLC, with the method’s precision, stability, and repeatability thoroughly evaluated. Subsequently, the effects of the 15 batches of KXS were assessed in an olfactory escape memory experiment, utilizing Aβ_42_ transgenic *drosophila* as a model. Finally, the spectrum-effect relationship between the KXS fingerprint and memory improvement was analyzed, with the active ingredients subjected to validation testing.

**Results:**

The results identified seventeen common peaks in the fingerprint, and eight active components were determined: polygalaxanthone III, 3-6-disinapoylsucrose, ginsenoside Rg1, ginsenoside Rb1, β-asarone, α-asarone, dehydrotumulosic acid, and dehydropachymic acid. Treatment with KXS (1%, for 4 days) significantly enhanced the performance index of Aβ_42_ flies in the olfactory experiment. Both spectrum-effect analysis and validation tests indicated that polygalaxanthone III, ginsenoside Rg1, ginsenoside Rb1, β-asarone, and α-asarone were positively correlated with the performance index and improved the performance index in the olfactory experiment. The HPLC fingerprint method for KXS demonstrated excellent precision, accuracy, and reproducibility, making it suitable for quality evaluation and control of KXS. Polygalaxanthone III, ginsenoside Rg1, ginsenoside Rb1, β-asarone, and α-asarone are identified as potential active ingredients of KXS for anti-AD effects.

**Discussion:**

These findings provide an experimental basis for developing new drugs based on KXS and its active ingredient combinations.

## Graphical Abstract

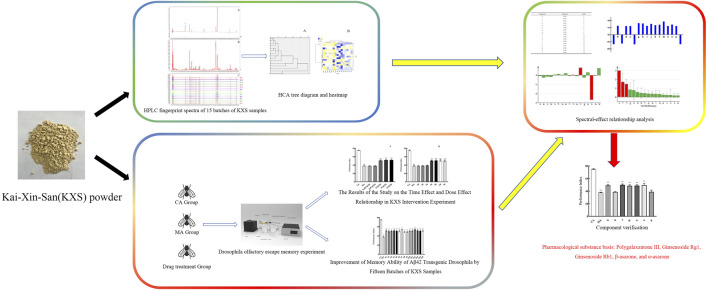

## 1 Introduction

Alzheimer’s disease (AD), a degenerative disorder of the central nervous system, is characterized by its insidious onset and age-related progression (Liu et al., 2024b). Clinically, the hallmark features of AD include memory decline and cognitive impairment (Huang et al., 2024). The incidence of AD increases significantly with age; among individuals over 65 years old, the incidence ranges from 4% to 7%, while for those over 85, it rises dramatically by 20%–30% (Lopez-Lee et al., 2024). According to the International Alzheimer’s Association, globally, a new case of AD is diagnosed every 3 s, underscoring its status as a major public health concern with severe implications for human wellbeing (Liu et al., 2024a). This highlights the urgent need to develop effective treatments for AD.

The pathological mechanisms underlying AD are highly complex, and its precise etiology remains unclear. Aβ and tau proteins are pivotal in the disease’s pathogenesis. Additionally, chronic inflammation, mitochondrial dysfunction, oxidative stress-induced neuronal damage or apoptosis, and other factors contribute significantly to the development of AD (Bero et al., 2011; Li et al., 2011; Ma et al., 2011; Dong et al., 2022; Kim et al., 2024). Current therapeutic drugs show limited efficacy, often targeting only one or a few elements of the disease’s pathological processes, such as reducing amyloid-protein deposition (Tabor and Holtzman, 2023) or inhibiting neuronal apoptosis (Xu et al., 2021). However, these treatments do not effectively halt disease progression. Moreover, medications like acetylcholinesterase inhibitors (e.g., donepezil) and NMDA receptor antagonists (e.g., memantine) can cause side effects, including nausea, vomiting, and cardiac arrhythmia (Buck et al., 2024; Egunlusi and Joubert, 2024).

*Drosophila melanogaster* is widely utilized as a genetic model organism due to its short life cycle, rapid reproduction, and well-mapped genome. Numerous AD models have been established by expressing AD-related proteins in the brains of flies. Aβ_42_ transgenic *drosophila*, which expresses the human Aβ_42_ protein in its brain, exhibits reduced learning and memory capabilities, a shortened lifespan, and degenerative changes in brain regions (Iijima-Ando and Iijima, 2010). This model serves as a powerful tool for studying Aβ_42_ and evaluating potential anti-AD drugs.

Traditional Chinese medicine (TCM) achieves therapeutic effects through multi-target and multi-perspective interventions, enabling comprehensive regulation and modulation of complex disease mechanisms. Compared to chemical drugs, TCM generally exhibits better tolerance and lower toxicity, offering significant therapeutic advantages in treating complex chronic diseases like AD (Ding et al., 2022). Kai-Xin-San (KXS), originally recorded in Bei Ji Qian Jin Yao Fang by Sun Simiao during the Tang Dynasty, is primarily used for treating mental restlessness, amnesia, insomnia, and severe palpitations (Yan et al., 2024). Modern pharmacological studies have demonstrated that KXS possesses anti-AD (Xu et al., 2023), antidepressant (Wang Y. T. et al., 2024), anti-inflammatory (Bai et al., 2022), and sleep-disorder-improving properties. Its anti-AD effects are achieved through various mechanisms, such as reducing amyloid-β (Aβ) protein deposition (Wang H. et al., 2024), upregulating protein kinase B phosphorylation levels (Jiao et al., 2022), improving mitochondrial dysfunction (Wang et al., 2023), and exerting neuroprotective effects (Zhang et al., 2023). The four herbal ingredients in KXS have also been studied for their anti-dementia properties. Research indicates that the primary active components with anti-AD effects include saponins in ginseng and polygala, oligosaccharide esters in polygala, phenylpropanoid compounds such as asarone in acorus gramineus, and triterpenic acids in poria (Park et al., 2019; Meng et al., 2021; Yuan et al., 2021; Zeng et al., 2021; Xu et al., 2023). However, the chemical complexity of KXS and the variability in medicinal efficacy due to factors such as origin, storage, and harvest season present challenges for quality consistency and reproducibility.

Evaluating the quality of TCM requires comprehensive, macroscopic, and non-linear analytical approaches. The HPLC fingerprint spectrum, as a quality control method, meets this requirement effectively (Xu et al., 2022). However, HPLC fingerprint does not directly associate these components with therapeutic efficacy. Establishing a spectrum-effect relationship to connect fingerprint profiles with drug efficacy is essential for elucidating the pharmacodynamic material basis of TCM and developing effective quality control methods. This approach integrates chemical composition, pharmacological action, and disease relevance (Xu et al., 2022).

In this study, the HPLC technique was utilized to develop a fingerprint profile for KXS. Using an Aβ_42_ transgenic *drosophila* model and memory performance as indicators, the spectrum-effect relationship was analyzed through grey relational analysis, bivariate correlation analysis, and partial least-squares regression. This analysis clarified the pharmacodynamic material basis of KXS, established quality standards, and ensured consistency in quality and therapeutic efficacy. Furthermore, these findings provide a solid foundation for the development of new drugs and the optimization of preparation techniques.

## 2 Materials and methods

### 2.1 Composition, source, and preparation of KXS sample solution

KXS is composed of ginseng, polygala, poria and acorus gramineus, with a proportion of 1:1:2:1, according to the Notice by the National Administration of Traditional Chinese Medicine of China.

Ginseng is the dried root and rhizome of *Panax ginseng* C. A. Mey. of the Araliaceae family. Polygala is the dried root of *Polygala tenuifolia* Willd. of the Polygalaceae family. Poria is the dried sclerotium of *Poria cocos* (Schw.) Wolf, a fungus of the Polyporaceae family. Acorus is the dried rhizome of *Acorus tatarinowii* Schott of the Araceae family. The above-mentioned medicinal materials were all provided by Shineway Pharmaceutical Group Co., Ltd., with a total of 15 batches (S1-S15).

Accurately weigh 1 g of KXS, add 5 mL of 95% methanol, and record the weight. Heat the mixture under reflux for 30 min, then allow it to cool. Re-weigh the solution and adjust to the original weight using 95% methanol. Mix thoroughly, filter, and collect the filtrate for use.

### 2.2 Preparation of chemicals and reference solutions

Polygalaxanthone III (DST230425-089), 3-6-disinapoylsucrose (DST210510-022), ginsenoside Rg1 (DSTDR000902), ginsenoside Rb1 (DSTDR000603), β-asarone (DSTDA001901), α-asarone (DST201025-044), dehydrotumulosic acid (DSTDQ010202), dehydropachymic acid (DSTDQ010101) were purchased from Dest Biotechnology Co., Ltd. (Sichuan, China). Acetonitrile (20240701, TEDIA) and methanol (20240628, TEDIA) were obtained from DiMa Technology Co., Ltd. (Beijing, China).

Dissolve an appropriate amount of the relevant components in 95% methanol to prepare the test solution. The concentrations of polygalaxanthone III, 3-6-disinapoylsucrose, ginsenoside Rg1, ginsenoside Rb1, β-asarone, α-asarone, dehydrotumulosic acid and dehydropachymic acid are 0.74 mg. mL^−1^, 0.76 mg. mL^−1^, 0.59 mg. mL^−1^, 0.35 mg. mL^−1^, 0.85 mg. mL^−1^, 1.42 mg. mL^−1^, 0.61 mg. mL^−1^ and 0.58 mg. mL^−1^, respectively.

### 2.3 *Drosophila* strains, sources, and rearing conditions

Wild type W^1118^, Elav-Gal4, and UAS-Aβ_42_ *drosophila* were all provided by the Core Facility of *Drosophila* Resource and Technology, CEMCS, CAS.

Main groups: I Control group with the same genetic background (CA): Select male *drosophila* from the first generation of Elav-Gal4 virgin *drosophila* and W^1118^ male *drosophila* hybrid offspring. II Aβ_42_ group (MA): Red-eyed straight-winged males were selected from the progeny generation of Elav-Gal4 virgin *drosophila* and UAS-Aβ_42_ male *drosophila* (Elav/Y; UAS-Aβ/+, Aβ_42_ *drosophila*) (Tue et al., 2020).

Standard *drosophila* Medium: The flies culture medium consists of cornmeal, soybean flour, sucrose, maltose, agar, yeast, and water. Specifically, it contains 6.7 g of cornmeal, 0.8 g of soybean flour, 4.0 g of sucrose, 4.2 g of maltose, 1.0 g of agar, 2.5 g of yeast, and 100 mL of water. Besides, sodium benzoate, with a dosage of 0.1 g, is selected as the preservative. The flies were maintained at a temperature of 25°C, with 60% humidity, under a 12-h light and 12-h dark cycle. The medium was refreshed every 3–4 days for passaged flies.

KXS-Containing Medium: Following the method for preparing Chinese medicine-containing medium described in previous research (Zhang et al., 2016), the KXS-containing medium was prepared with concentrations ranging from 0.25% to 4%. Before each administration, the *drosophila* were fasted for 3 h.

### 2.4 Analysis conditions of HPLC

The HPLC chromatographic analysis was performed using a Waters e2695 HPLC-DAD system (Waters, United States) equipped with a Waters Symmetry C_18_ chromatographic column (4.6 × 250 mm, 5 μm) (Waters, United States). For gradient elution, the mobile phases consisted of acetonitrile solution (organic phase C) and 0.2% phosphoric acid aqueous solution (organic phase D), with a flow rate of 1 mL min^−1^. The temperature of the chromatographic column was maintained at 30°C. The injection volume was set to 20 μL, and the chromatograms were detected at a wavelength of 203 nm. The gradient elution program was as follows: 0–2 min, 10–12%C; 2–10 min, 12–14%C; 10–14 min, 14–15%C; 14–21 min, 15–16%C; 21–23 min, 16–18%C; 23–33 min, 18–26%C; 33–48 min, 26–30%C; 48–58 min, 30–35%C; 58–68 min, 35–37%C; 68–78 min, 37%C; 78–93 min, 37–50%C; 93–103 min, 50–74%C; 103–115 min, 74–82%C; 115–120 min, 82–10%C.

### 2.5 HPLC method validation

The KXS sample test solution (S1) was prepared following the procedure described in Section 2.1. Six consecutive injections were performed under the conditions outlined in Section 2.4 to assess the precision of the instrument. The S1 test solution was injected at intervals of 0, 2, 4, 8, 12, and 24 h to evaluate sample stability. Additionally, six separate test solutions of S1 samples were prepared and injected to assess sample reproducibility.

### 2.6 Chromatographic peak identification

The reference solution, prepared as described in Section 2.2, was analyzed using the chromatographic system. The retention time of the reference peak was recorded, and the retention times of the reference components in the sample solution were determined by comparison with the recorded reference peak retention times.

### 2.7 Fingerprint similarity analysis

Fifteen batches of KXS samples were analyzed using the standardized HPLC method. The resulting chromatographic data were exported in CDF format and processed using the Similarity Evaluation System for Chromatographic Fingerprints of Traditional Chinese Medicines (version 2012).

### 2.8 Hierarchical cluster analysis

Systematic cluster analysis was performed using SPSS statistical software (IBM, United States). The between-groups linkage method was employed to analyze the chromatographic peak area data from the fingerprint spectra of 15 batches of KXS. The similarity between samples was measured using the Euclidean distance.

### 2.9 Behavioral experiments

#### 2.9.1 *Drosophila* olfactory escape memory experiment

The experimental apparatus was provided by SANS Biotechnology Co., Ltd. (Supplementary Figure S1). The experiments were conducted in an environment maintained at a constant temperature of 25°C and humidity of 60%, under dark infrared conditions.

The olfactory escape memory experiment consisted of two phases: the training phase and the testing phase (Yu et al., 2024). In the training phase, a four-step process was followed. First, 100 flies were placed into the training tubes to adapt for at least 90 s. Second, the flies were exposed to CS + gas (4-methylcyclohexanol, MCH, Sigma, 104191) and synchronously subjected to 1.5-s pulses of 60 V electric shocks delivered every 3.5 s for a total of 12 pulses. Third, fresh air was introduced for 45 s to clear any residual CS + gas. Finally, the flies were exposed to CS- gas (3-octanol, OCT, Sigma, 218405) for 1 min without electric shocks. After the training phase, the testing phase was conducted immediately to examine the flies’ instantaneous memory. Flies that had undergone the training phase were moved to the choice point of the T-maze, which contained two arms exposed to CS + gas and CS- gas, respectively. The flies were allowed to choose between the two arms based on olfactory cues within 2 min. Afterward, the flies were anesthetized with CO_2_, and the numbers of flies in the CS + gas and CS- gas arms were recorded.

The performance index (PI) was used to evaluate the memory performance of flies. The calculation formula is as follows:

$$PI=\left[Number\left(CS-\right)-Number\left(CS+\right)\right]÷\left[Number\left(CS-\right)+Number\left(CS+\right)\right]\times 100$$

The average value of PI was calculated from the PI of fourteen batches flies in olfactory escape memory experiment (n = 14).

#### 2.9.2 Investigation of time-effect and dose-effect relationships in KXS intervention experiments

Using the experimental method described in “2.9.1,” an investigation was conducted to determine the effective dosage concentration and administration duration of KXS. Specifically, five concentrations of KXS—0.25%, 0.5%, 1%, 2%, and 4%—were examined. Administration at these concentrations was carried out for 4 days. Additionally, the study explored the effects of different durations of continuous dosing (1 day, 2 days, 3 days, 4 days, and 5 days) at a fixed concentration of 1% KXS.

#### 2.9.3 Improvement effect of different batches of KXS samples on memory ability

The Aβ_42_ transgenic *drosophila* were divided into fifteen groups, and each group received drugs from a specific batch. Following the experimental method outlined in “2.9.1,” the number of *drosophila* in the respective gas environments was recorded for each group, and the PI for memory was calculated.

### 2.10 Spectrum-effect relationship analysis

The spectrum-effect relationship, which refers to the connection between the peak areas of the common peaks in KXS and its effect on enhancing cognitive ability, was established using grey relational analysis, bivariate correlation analysis, and partial least squares regression analysis.

### 2.11 Statistical analysis

Data are presented as mean ± SD. SPSS was used for statistical analysis, and GraphPad (United States) was employed for graphing. One-way ANOVA was conducted for data analysis. Statistical significance was indicated by *P* < 0.05.

## 3 Results

### 3.1 HPLC method validation

Using the α-asarone peak (peak 0) as the reference, the relative standard deviation (RSD) for precision ranged from 0% to 0.6%, for reproducibility from 0.1% to 0.5%, and for stability from 0% to 1.3%. The results showed that the RSD of the relative peak area of each chromatographic peak in the fingerprint chromatogram was less than 2%. The HPLC method demonstrated excellent accuracy, and the KXS sample solution remained stable for up to 24 h. These findings confirm that the developed HPLC fingerprint method has superior reproducibility.

### 3.2 Establishment of fingerprint spectra for fifteen batches of KXS

**FIGURE 1:**
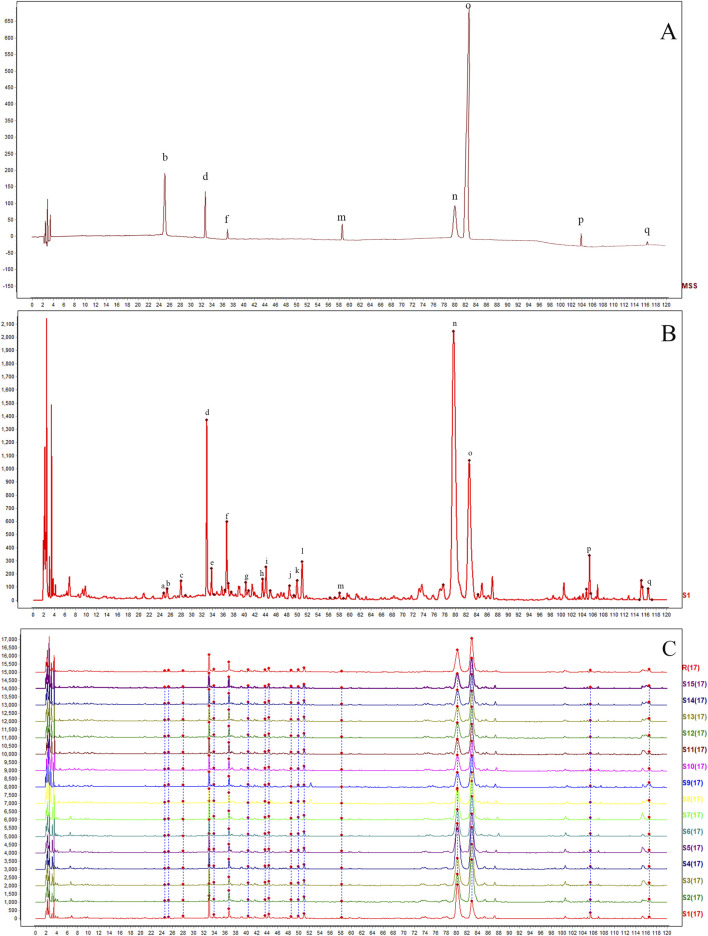
HPLC fingerprints of 15 batches of Kai-Xin-San **(A)** Mixed reference substance solution, **(B)** KXS sample and **(C)** 15 batches of KXS. b: polygalaxanthone III; d: 3-6-disinapoylsucrose; f: ginsenoside Rg1; m: ginsenoside Rb1; n: β-asarone; o: α-asarone; p: dehydrotumulosic acid; q: dehydropachymic acid.

As illustrated in Figure 1, chromatograms were obtained for the mixed reference solution (Figure 1A), the KXS test samples (Figure 1B), and each batch of KXS (Figure 1C) under the specified chromatographic conditions. By comparing the ultraviolet spectra and the retention times of the reference substance peaks, 17 common peaks were identified within the 0–120 min range. Eight components were identified: polygalaxanthone III (b), 3-6-disinapoylsucrose (d), ginsenoside Rg1 (f), ginsenoside Rb1 (m), β-asarone (n), α-asarone (o), dehydrotumulosic acid (p), and dehydropachymic acid (q). Peak o, with the largest area in the fingerprint spectrum, was designated as the characteristic peak following chromatographic system analysis. The relative peak areas of each common peak are listed in Table 1, relative retention times in Table 2, and the proportion of non-common peak areas in Table 3. Variations in the RSD of relative peak areas reflect differences in the compound content among batches.

**TABLE 1:** The relative peak areas of 17 common peaks in HPLC fingerprints of Kai-Xin-San.

Sample	a	b	c	d	e	f	g	h	i	j	k	l	m	n	o	p	q
S1	0.01	0.03	0.02	0.17	0.02	0.09	0.02	0.03	0.05	0.03	0.04	0.07	0.01	0.63	1.00	0.03	0.05
S2	0.02	0.03	0.02	0.17	0.02	0.09	0.02	0.03	0.05	0.02	0.04	0.07	0.01	0.62	1.00	0.03	0.05
S3	0.01	0.03	0.02	0.17	0.02	0.09	0.02	0.03	0.05	0.04	0.04	0.07	0.01	0.63	1.00	0.03	0.04
S4	0.01	0.03	0.02	0.17	0.02	0.09	0.02	0.03	0.04	0.04	0.04	0.07	0.02	0.62	1.00	0.02	0.05
S5	0.01	0.03	0.02	0.17	0.02	0.09	0.02	0.03	0.05	0.03	0.04	0.06	0.02	0.63	1.00	0.03	0.04
S6	0.01	0.03	0.02	0.17	0.02	0.09	0.02	0.03	0.05	0.03	0.04	0.06	0.02	0.63	1.00	0.03	0.03
S7	0.01	0.03	0.01	0.16	0.02	0.08	0.02	0.03	0.04	0.03	0.04	0.06	0.02	0.58	1.00	0.03	0.03
S8	0.02	0.03	0.02	0.17	0.02	0.09	0.02	0.02	0.05	0.01	0.04	0.03	0.00	0.63	1.00	0.03	0.04
S9	0.02	0.03	0.02	0.17	0.02	0.09	0.02	0.03	0.05	0.01	0.04	0.03	0.01	0.63	1.00	0.03	0.04
S10	0.02	0.03	0.01	0.15	0.02	0.08	0.02	0.03	0.04	0.03	0.04	0.06	0.01	0.56	1.00	0.02	0.03
S11	0.02	0.03	0.01	0.17	0.02	0.08	0.02	0.03	0.04	0.03	0.04	0.06	0.01	0.58	1.00	0.03	0.04
S12	0.02	0.03	0.01	0.16	0.02	0.08	0.02	0.03	0.04	0.03	0.04	0.06	0.01	0.59	1.00	0.03	0.03
S13	0.01	0.03	0.02	0.16	0.02	0.08	0.02	0.03	0.04	0.03	0.04	0.06	0.01	0.59	1.00	0.03	0.04
S14	0.02	0.03	0.02	0.16	0.02	0.08	0.02	0.03	0.04	0.03	0.04	0.06	0.01	0.59	1.00	0.03	0.04
S15	0.02	0.03	0.02	0.16	0.02	0.08	0.02	0.03	0.04	0.03	0.04	0.06	0.01	0.59	1.00	0.03	0.04
RSD (%)	22.42	3.60	9.03	3.95	4.21	5.91	4.37	7.01	3.73	32.02	4.20	19.96	30.91	4.12	0.00	2.86	15.44

Note: The o-peak is regarded as the characteristic peak.

**TABLE 2:** The relative retention times of 17 common peaks in HPLC fingerprints of Kai-Xin-San.

Sample	a	b	c	d	e	f	g	h	i	j	k	l	m	n	o	p	q
S1	0.29	0.30	0.35	0.41	0.42	0.46	0.50	0.54	0.55	0.60	0.62	0.64	0.72	1.03	1.00	1.32	1.46
S2	0.29	0.30	0.35	0.41	0.42	0.45	0.50	0.54	0.55	0.60	0.63	0.63	0.72	1.03	1.00	1.31	1.45
S3	0.30	0.30	0.35	0.41	0.41	0.45	0.50	0.54	0.55	0.60	0.63	0.63	0.72	1.03	1.00	1.32	1.45
S4	0.29	0.29	0.34	0.40	0.42	0.45	0.50	0.54	0.54	0.60	0.61	0.63	0.72	1.03	1.00	1.31	1.44
S5	0.30	0.31	0.35	0.41	0.41	0.46	0.50	0.54	0.55	0.60	0.62	0.62	0.72	1.03	1.00	1.32	1.46
S6	0.29	0.30	0.35	0.41	0.42	0.45	0.50	0.54	0.55	0.60	0.62	0.63	0.72	1.03	1.00	1.31	1.45
S7	0.29	0.30	0.35	0.41	0.42	0.46	0.50	0.54	0.55	0.61	0.62	0.64	0.73	1.04	1.00	1.33	1.46
S8	0.29	0.30	0.35	0.41	0.42	0.46	0.51	0.55	0.55	0.61	0.63	0.64	0.73	1.03	1.00	1.33	1.47
S9	0.29	0.30	0.35	0.41	0.42	0.46	0.50	0.54	0.55	0.61	0.63	0.64	0.73	1.04	1.00	1.33	1.47
S10	0.29	0.30	0.35	0.41	0.42	0.46	0.51	0.55	0.55	0.61	0.62	0.64	0.73	1.04	1.00	1.33	1.47
S11	0.29	0.30	0.34	0.41	0.42	0.46	0.50	0.54	0.55	0.61	0.62	0.64	0.73	1.03	1.00	1.32	1.46
S12	0.29	0.30	0.35	0.41	0.42	0.46	0.50	0.54	0.55	0.61	0.63	0.64	0.73	1.04	1.00	1.33	1.47
S13	0.29	0.30	0.35	0.41	0.42	0.46	0.50	0.54	0.55	0.61	0.62	0.64	0.73	1.05	1.00	1.33	1.46
S14	0.29	0.30	0.35	0.41	0.42	0.46	0.50	0.54	0.55	0.61	0.62	0.64	0.73	1.04	1.00	1.32	1.46
S15	0.29	0.30	0.34	0.41	0.42	0.46	0.50	0.54	0.55	0.60	0.62	0.63	0.73	1.05	1.00	1.32	1.46
RSD (%)	0.80	1.10	0.30	0.40	0.80	0.50	0.60	0.60	0.50	0.50	0.80	0.70	0.70	0.70	0.00	0.50	0.60

Note: The o-peak is regarded as the characteristic peak.

**TABLE 3:** The non-common peak area ratio of HPLC fingerprint of 15 batches of Kai-Xin-San.

Sample	Rate (%)	Sample	Rate (%)	Sample	Rate (%)
S1	4.40	S6	6.77	S11	5.55
S2	6.97	S7	8.33	S12	5.56
S3	5.34	S8	8.40	S13	5.75
S4	6.05	S9	8.58	S14	6.12
S5	7.44	S10	8.77	S15	6.64

### 3.3 Fingerprint similarity analysis

To assess sample similarity, a comparative analysis was performed between the fingerprint chromatograms of the 15 KXS batches and a reference chromatogram. The similarity range for the batches compared to the reference fingerprint was between 0.887 and 1, as detailed in Table 4. Most samples exhibited high similarity, with values close to or exceeding 0.99. For example, batches S2, S3, S6, and S7 showed similarities above 0.99, while S5 reached 1, indicating consistent chemical composition and relative content among these batches. This suggests stable quality and a well-controlled production process. However, batch S1, with a similarity of 0.887, displayed relatively lower similarity. This may point to minor differences in raw materials, processing, or storage, causing slight variations in chemical components. Despite this, the similarity remained high overall, indicating that the deviations were within an acceptable range.

**TABLE 4:** The similarity of HPLC fingerprint of 15 batches of Kai-Xin-San.

Sample	Similarities	Sample	Similarities	Sample	Similarities
S1	0.887	S6	0.998	S11	0.991
S2	0.999	S7	0.999	S12	0.991
S3	0.999	S8	0.997	S13	0.991
S4	0.98	S9	0.994	S14	0.991
S5	1	S10	0.989	S15	0.992

### 3.4 Hierarchical clustering analysis (HCA)

**FIGURE 2:**
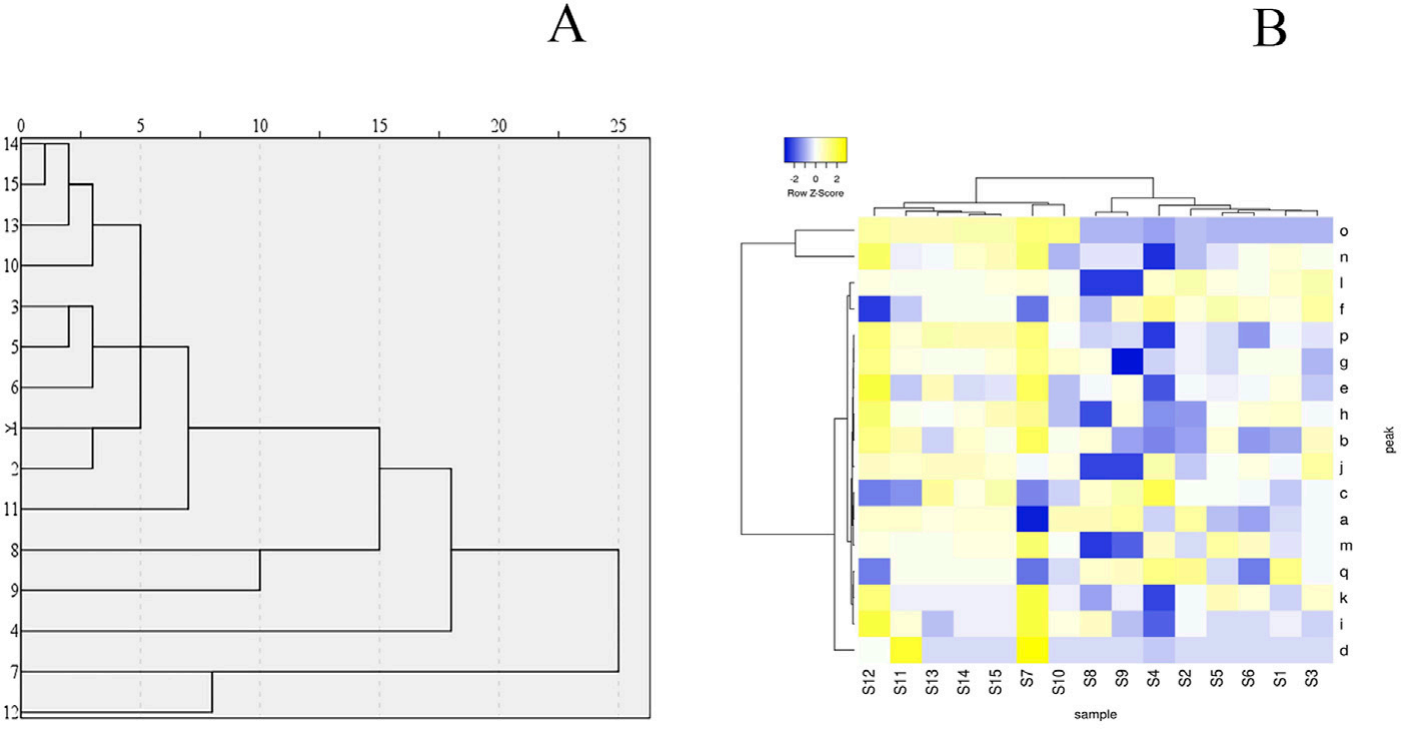
The hierarchical clustering analysis for common peak areas in Kai-Xin-San HPLC fingerprint **(A)** Dendrogram and **(B)** heat map.

The study employed the HCA function in SPSS statistical software (IBM, USA) and used the inter-group linkage method to analyze the fingerprints of 15 batches of KXS. HCA was applied to assess the correlation of the KXS HPLC fingerprints, and testing was performed using Heatmapper (http://www.heatmapper.ca/) (Xu et al., 2022). The HCA dendrogram (Figure 2A) and heatmap (Figure 2B) were generated. Each color unit in the heatmap corresponds to a matrix representing a numerical value. As seen in Figure 2, four clusters were identified. S1, S2, S3, S5, S6, S10, S11, S13, S14, and S15 form one group; S9 and S8 form another group; S4 forms a separate group; and S12 and S7 form a final group. The HCA results indicate that the different batches of KXS samples share similar fingerprints, but the peak areas of the chemical components differ, suggesting variations in the content of the chemical components. HCA can effectively differentiate samples at the chemical level.

### 3.5 Results of the study on the time effect and dose effect relationship in KXS intervention experiment

**FIGURE 3:**
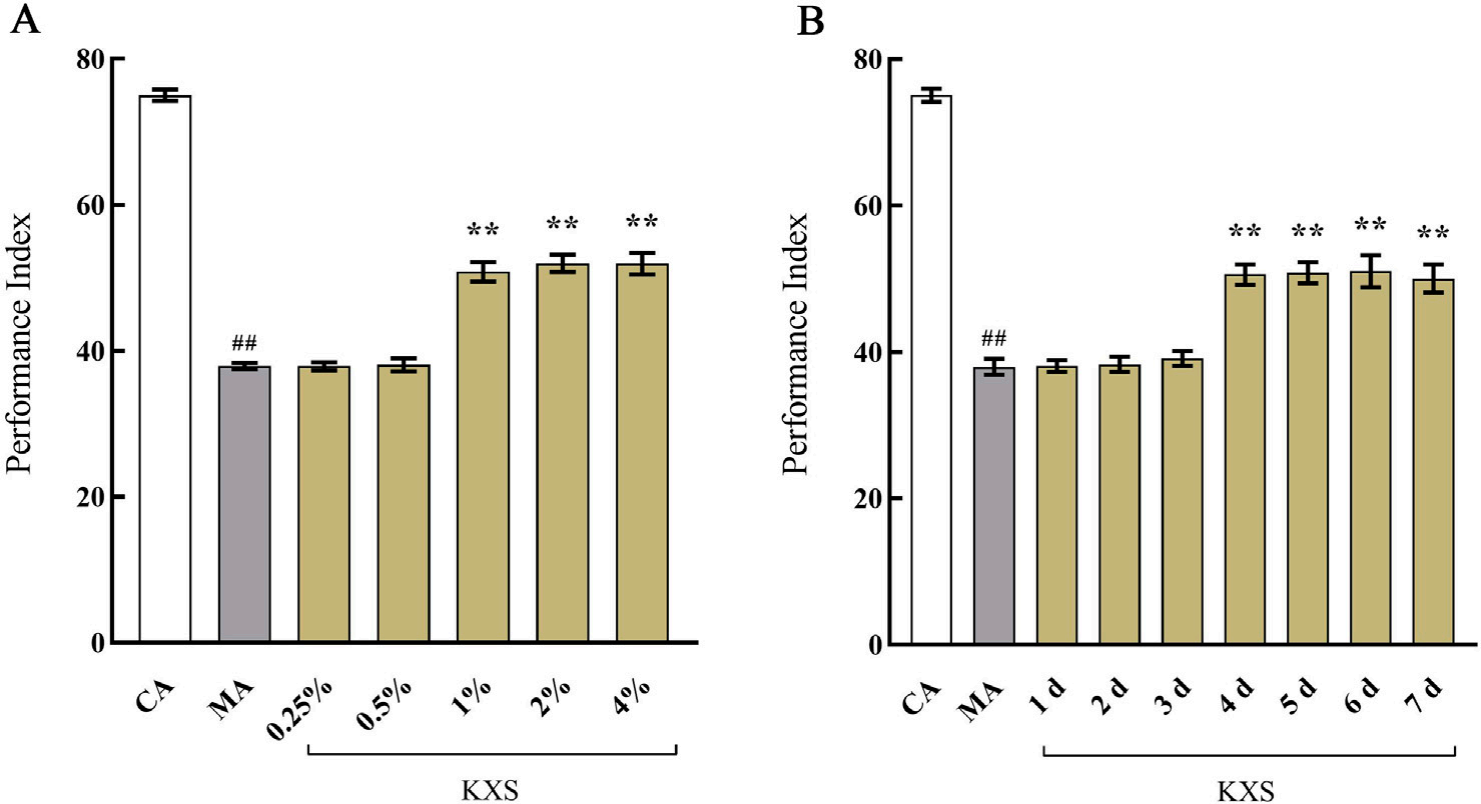
The effects of Kai-Xin-San on the performance index of I of Aβ_42_ transgenic *drosophila* **(A)** Different concentration and **(B)** different duration. CA: Control group with the same genetic background. MA: Aβ_42_ transgenic group. n = 14 per group. Data are presented as Mean ± SD. Compared with the CA, ^##^ *P* < 0.01; compared with the MA, **P < 0.01.

The experiments demonstrated that, except for the 0.25% and 0.5% KXS groups, which were ineffective, all other concentrations of KXS significantly enhanced the PI of Aβ_42_ transgenic *drosophila* (Figure 3A). Similarly, except for the groups treated for 1, 2, and 3 days, all other treatment duration groups also showed improvements in PI (Figure 3B). Based on these results, the optimal administration concentration was determined to be 1% KXS, with a treatment duration of 4 days.

### 3.6 Improvement of memory ability in Aβ_42_ transgenic *Drosophila* by fifteen batches of KXS samples

**FIGURE 4:**
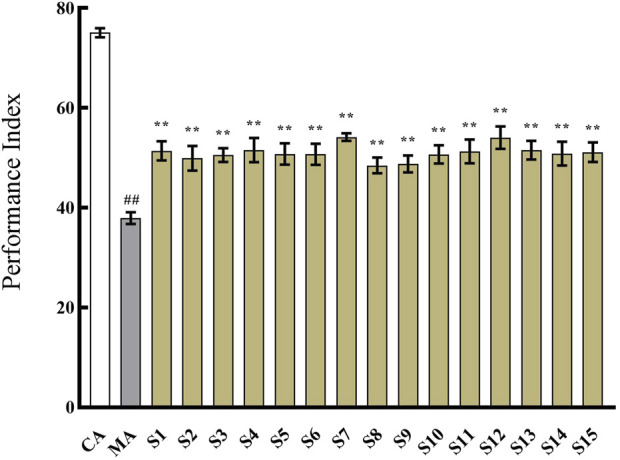
The effect of 15 batches of Kai-Xin-San on the performance index of Aβ_42_ transgenic *drosophila*. CA: Control group with the same genetic background. MA: Aβ_42_ transgenic group. S1∼S15: Kai-Xin-San samples S1∼S15. n = 14 per group. Data are presented as Mean ± SD. Compared with the CA, ^##^ *P* < 0.01; compared with the MA, ^**^ *P* < 0.01.

As shown in Figure 4, the 15 batches of KXS samples all demonstrated an enhancement effect on cognitive impairment in Aβ_42_ transgenic *drosophila*. However, there were variations among the different samples in the extent of cognitive improvement. This discrepancy may be attributed to the differences in the chemical composition of the samples. Therefore, the spectrum-effect relationship was further investigated.

### 3.7 Spectral effect relationship analysis

#### 3.7.1 Grey relational analysis (GRA)

GRA is a multifactorial statistical technique used to determine the strength of the association between each chemical constituent and drug efficacy. The correlation coefficient ranges from 0 to 1, with higher values indicating a stronger correlation with the improvement of cognitive impairment. A higher correlation coefficient suggests a stronger connection between the constituent and the drug efficacy, making it more likely to be a significant active substance. The chemical constituents with a correlation coefficient exceeding 0.8 are e (0.954), i (0.953), d (0.952), n (0.951), k (0.947), b (0.945), p (0.941), g (0.936), f (0.928), o (0.922), h (0.886), and c (0.862). These results suggest that these constituents may be potential active substances for the treatment of AD.

#### 3.7.2 Bivariate correlation analysis (BCA)

**FIGURE 5:**
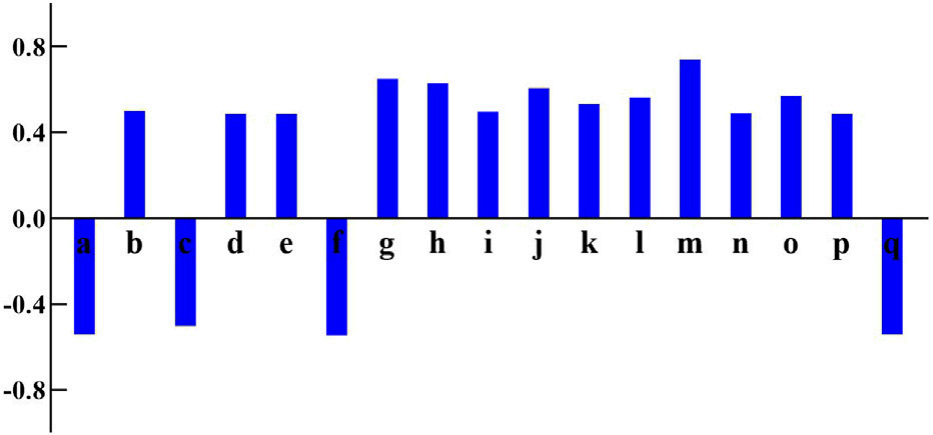
The correlation coefficients between peaks area and performance index of 17 common peaks in Kai-Xin-San HPLC fingerprints.

The purpose of BCA is to examine the linear association between two variables. The pearson correlation coefficient is used to measure the degree of correlation between elements and drug efficacy. The higher the absolute value of this coefficient, the stronger the correlation. The data used is both reliable and representative. In this study, the BCA tool in SPSS statistical software (IBM, United States) was utilized to evaluate the correlation between the chromatographic peak area values and the PI. As shown in Figure 5, the components m, g, h, j, l, o, b, i, n, k, d, e, and p show a positive correlation with the cognitive improvement effect and may be potential active components for the treatment of AD.

#### 3.7.3 Partial least - Squares regression (PLSR)

**FIGURE 6:**
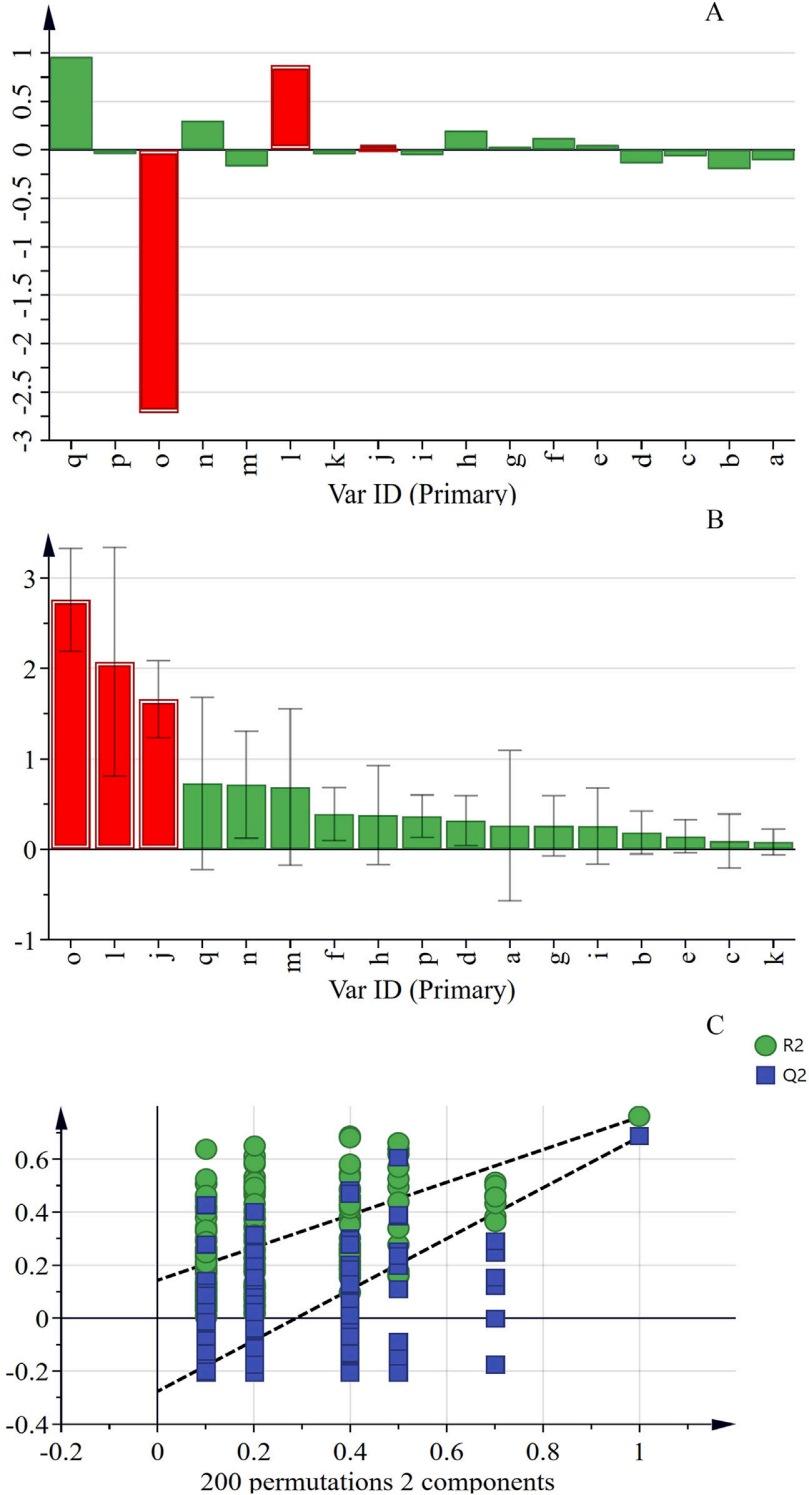
The PLSR analysis between peaks area and performance index of 17 common peaks in Kai-Xin-San HPLC fingerprints **(A)** Coefficients, **(B)** VIP **(C)** PLSR model.

In order to investigate the comprehensive influence of peak areas on the PI of flies, PLSR analysis was conducted using software SIMCA-14.1 (Sartorius Stedim, Sweden). As shown in Figures 6A, B, in this model, when R^2^ and Q^2^ are both greater than 0.5, it indicates that the model not only fits the data well but also has a certain degree of accuracy in predicting new data, demonstrating good generalization ability. Thus, the model is considered to have a favorable fit. Using a VIP value greater than 1 as the criterion for screening potential active components, when the VIP value exceeds 1, the chemical component contributes significantly to the model. Additionally, 200 permutation tests were conducted. The intersection point of the Q^2^ regression line and the y-axis is located on the negative semi-axis, indicating that the model is not over-fitted and that the model validation result is valid (see Figure 6C). The results show that the VIP values of o, l, and j are all greater than 1, suggesting that these three components are related to the improvement of cognitive impairment. Therefore, these components may be potential active ingredients of KXS in the treatment of AD.

### 3.8 Component verification

**FIGURE 7:**
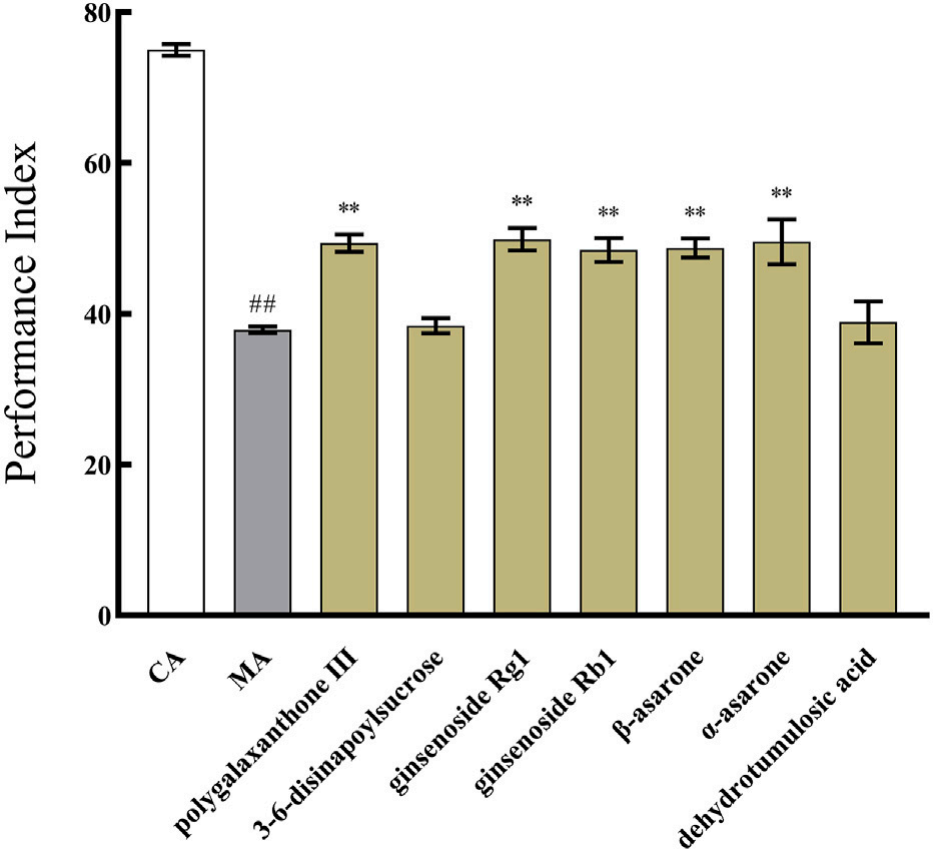
The effect of identified seven components of Kai-Xin-San on the performance index of Aβ_42_ transgenic *drosophila*. CA: Control group with the same genetic background. MA: Aβ_42_ transgenic group. n = 14 per group. Data are presented as Mean ± SD. Compared with the CA, ^##^ *P* < 0.01; compared with the MA, ^**^ *P* < 0.01.

Based on the results of the above spectrum-effect relationship analysis, b, c, d, e, f, g, h, i, j, k, l, m, n, o, and p may be potential active ingredients of KXS. Among these, seven identified ingredients were selected for subsequent verification to further analyze the impact of a single ingredient on the PI of Aβ_42_ transgenic *drosophila*. As described in Figure 7, the PI values of the 15 batches of KXS increased by an average of 35% compared to the MA. However, the PI of polygalaxanthone III, ginsenoside Rg1, ginsenoside Rb1, β-asarone, and α-asarone increased by 30.26%, 31.60%, 27.36%, 28.55%, and 30.74%, respectively. Compared to KXS, the PI growth rate of these active ingredients decreased to varying degrees.

## 4 Discussion

In our study, the HPLC fingerprint of KXS was successfully established, demonstrating good separation and repeatability. Eight chemical components were identified, which were derived from the four Chinese medicinal herbs constituting KXS. Polygalaxanthone III, ginsenoside Rg1, ginsenoside Rb1, β-asarone, and α-asarone may be the potential active ingredients of KXS in the treatment of AD. These results can serve as a foundation for controlling the quality standards of KXS on one hand and provide an experimental basis for the development of new drugs combining KXS and its active ingredients on the other hand.

Due to the complex composition of traditional Chinese medicine, the presence of more common peaks in its fingerprint spectra allows for more comprehensive quality control. Previous studies (Lv et al., 2016; Lin et al., 2021; Yin et al., 2021; Yang et al., 2022; Shan et al., 2023) established the fingerprint spectra of KXS, but the number of common peaks and the identification of components were relatively limited, failing to comprehensively reflect the characteristics of its chemical components. In contrast, the fingerprint spectra established in our study identified seventeen common peaks and eight components, effectively controlling the internal quality of KXS in a more comprehensive and systematic manner. Furthermore, the eight identified components all originated from the four Chinese medicinal herbs constituting KXS. Unlike previous studies, the active ingredients identified did not include those from ginseng (Lv et al., 2016) or poria cocos (Shan et al., 2023). As previously reported (Lv et al., 2016; Yang et al., 2022; Shan et al., 2023; Shang et al., 2024), our findings further confirm that the growth environment of the medicinal materials used in KXS influences its quality.

The Aβ_42_ transgenic *drosophila* model has been widely utilized for screening potential drugs for the treatment of AD (Iijima et al., 2004). Our study found that KXS exhibited a dose-dependent anti-AD effect in Aβ_42_ transgenic *drosophila*. Unlike traditional preparation methods, where KXS is typically extracted using water and methanol in pharmacodynamic studies (Shan et al., 2023; Su et al., 2023; Shang et al., 2024; Yan et al., 2024), in our study, KXS powder was dissolved directly into the *drosophila* culture medium. This approach ensured consistency with the classical preparation method, allowing us to explore the efficacy of KXS in a genetically manipulable and short-life-cycle model.

The spectrum-effect relationship provides an effective strategy for identifying active substances in Chinese medicine formulas. The spectrum-effect relationship analysis of KXS demonstrated that seven identified components were associated with the anti-Aβ_42_ effect. Further component-verification experiments revealed that polygalaxanthone III, ginsenoside Rg1, ginsenoside Rb1, β-asarone, and α-asarone improved the memory abilities of Aβ_42_ transgenic *drosophila*. Previous studies have shown that polygalaxanthone III (Park et al., 2019; Deng et al., 2020; Du et al., 2022; Xie et al., 2023), β-asarone (Meng et al., 2021; Balakrishnan et al., 2022), α-asarone (Zeng et al., 2021; Venkatesan, 2022), ginsenoside Rg1 (Sun et al., 2022; Wang et al., 2023; Deng et al., 2024), and ginsenoside Rb1 (Yang et al., 2020; Changhong et al., 2021; Yuan et al., 2021; Shalaby et al., 2023) all exhibited anti-AD pharmacological activities. Despite previous studies suggesting that 3-6-disinapoylsucrose exhibited anti-AD effects (Yuan et al., 2021), our study failed to demonstrate such effects. This discrepancy may be attributed to variations in the animal model used, the drug preparation method, and the administered dosage.

Nevertheless, this study has certain limitations. Under the current experimental paradigm, it is challenging to precisely determine whether KXS and its components affect the encoding stage of learning, the formation or maintenance stage of short-term memory, or both. Therefore, it may be necessary to conduct more paradigm in olfactory escape memory experiment to study the effect of KXS on improving cognition. Although the *drosophila* model has advantages in studying certain basic neurobiological processes, it has limitations in exploring long-term memory and differentiating between various memory systems. To more comprehensively evaluate the impact of KXS and its components on long-term memory, future research could employ rodent models for multi-stage and multi-type memory tests.

## 5 Conclusion

This study successfully established the KXS fingerprint method, which demonstrated precision, stability, and reproducibility. At the same time, the study also identified the key active ingredients of KXS that play an anti-AD role: polygalaxanthone III, ginsenoside Rg1, ginsenoside Rb1, β-asarone, and α-asarone. As a classic prescription in traditional Chinese medicine, KXS holds significant research potential, provided that quality is comprehensively monitored and effectively controlled. On the one hand, based on the pathological mechanism of AD, it is recommended to conduct further research into the anti-AD mechanism of KXS to provide a theoretical basis for its clinical application. On the other hand, the active ingredients of KXS can be combined to develop new drugs for the treatment of AD.

## Data Availability

The raw data supporting the conclusions of this article will be made available by the authors, without undue reservation.
