# Supplementary figures and images for: The spectrum-efficacy correlation of Kai-Xin-San for cognition of Aβ42 transgenic Drosophila and verification of its active ingredients
Source: Front Pharmacol. 2025 Jan 28;16:1538837. doi: 10.3389/fphar.2025.1538837 (PMC11811076; doi:10.3389/fphar.2025.1538837)

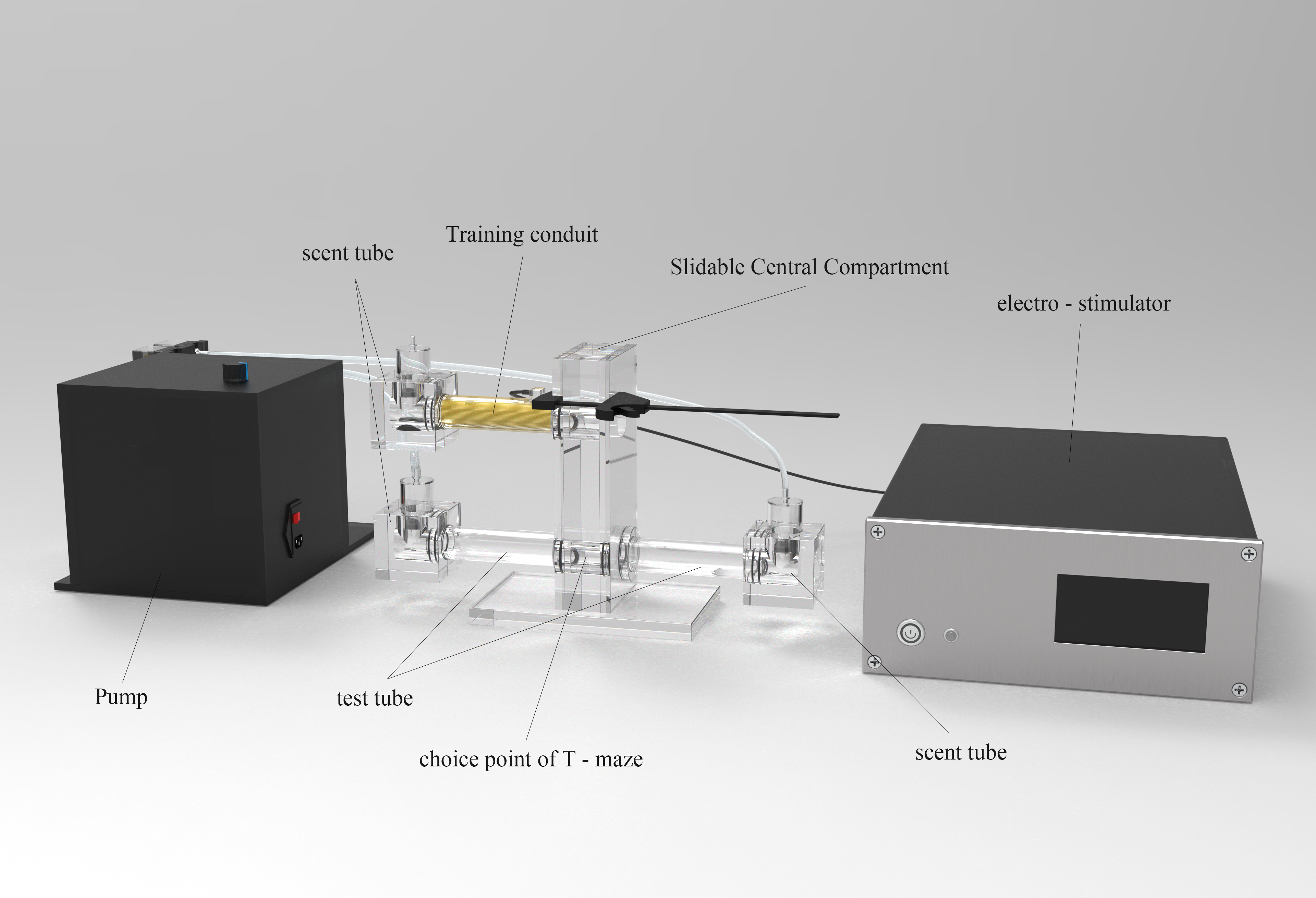

Supplement: Supplementary file 1 [file Image1.png]
